# Scaffold Hopping of α-Rubromycin Enables Direct Access to FDA-Approved Cromoglicic Acid as a SARS-CoV-2 M^Pro^ Inhibitor

**DOI:** 10.3390/ph14060541

**Published:** 2021-06-05

**Authors:** Hani A. Alhadrami, Ahmed M. Sayed, Heba Al-Khatabi, Nabil A. Alhakamy, Mostafa E. Rateb

**Affiliations:** 1 Department of Medical Laboratory Technology, Faculty of Applied Medical Sciences, King Abdulaziz University, P.O. Box 80402, Jeddah 21589, Saudi Arabia; hanialhadrami@kau.edu.sa (H.A.A.); halkhattabi@kau.edu.sa (H.A.-K.); 2 Molecular Diagnostic Lab, King Abdulaziz University Hospital, King Abdulaziz University, P.O. Box 80402, Jeddah 21589, Saudi Arabia; 3 Department of Pharmacognosy, Faculty of Pharmacy, Nahda University, Beni-Suef 62513, Egypt; Ahmed.mohamed.sayed@nub.edu.eg; 4 Center of Excellence in Genomic Medicine Research, King Abdulaziz University, Jeddah 21589, Saudi Arabia; 5 Department of Pharmaceutics, Faculty of Pharmacy, King Abdulaziz University, Jeddah 21589, Saudi Arabia; nalhakamy@kau.edu.sa; 6 School of Computing, Engineering & Physical Sciences, University of the West of Scotland, Paisley PA1 2BE, UK

**Keywords:** COVID-19, *Streptomyces collinus*, α-rubromycin, in silico, M^Pro^, cromolyn, cheminformatics

## Abstract

The COVID-19 pandemic is still active around the globe despite the newly introduced vaccines. Hence, finding effective medications or repurposing available ones could offer great help during this serious situation. During our anti-COVID-19 investigation of microbial natural products (MNPs), we came across α-rubromycin, an antibiotic derived from *Streptomyces collinus* ATCC19743, which was able to suppress the catalytic activity (IC_50_ = 5.4 µM and *K*_i_ = 3.22 µM) of one of the viral key enzymes (i.e., M^Pro^). However, it showed high cytotoxicity toward normal human fibroblasts (CC_50_ = 16.7 µM). To reduce the cytotoxicity of this microbial metabolite, we utilized a number of in silico tools (ensemble docking, molecular dynamics simulation, binding free energy calculation) to propose a novel scaffold having the main pharmacophoric features to inhibit M^Pro^ with better drug-like properties and reduced/minimal toxicity. Nevertheless, reaching this novel scaffold synthetically is a time-consuming process, particularly at this critical time. Instead, this scaffold was used as a template to explore similar molecules among the FDA-approved medications that share its main pharmacophoric features with the aid of pharmacophore-based virtual screening software. As a result, cromoglicic acid (*aka* cromolyn) was found to be the best hit, which, upon in vitro M^Pro^ testing, was 4.5 times more potent (IC_50_ = 1.1 µM and *K*_i_ = 0.68 µM) than α-rubromycin, with minimal cytotoxicity toward normal human fibroblasts (CC_50_ > 100 µM). This report highlights the potential of MNPs in providing unprecedented scaffolds with a wide range of therapeutic efficacy. It also revealed the importance of cheminformatics tools in speeding up the drug discovery process, which is extremely important in such a critical situation.

## 1. Introduction

The severe acute respiratory syndrome coronavirus 2 (SARS CoV-2) pandemic is still a serious global concern, and hope is hanging on vaccines to provide enough protection [1,2]. However, searching for suitable antiviral medications is also highly required to assist vaccines in containing this rapidly evolving infectious disease.

The SARS CoV-2 main protease (M^Pro^) is a key hydrolytic enzyme that can activate the viral polyprotein replication complex (1ab) by recognizing and cleaving its specific amino-acid sequences. Moreover, it is among the conserved proteins in the coronavirus family [3]. Hence, it has attracted many research groups and pharmaceutical companies developing specific and effective anti-SARS CoV-2 therapeutics [4,5].

**Figure 1:**
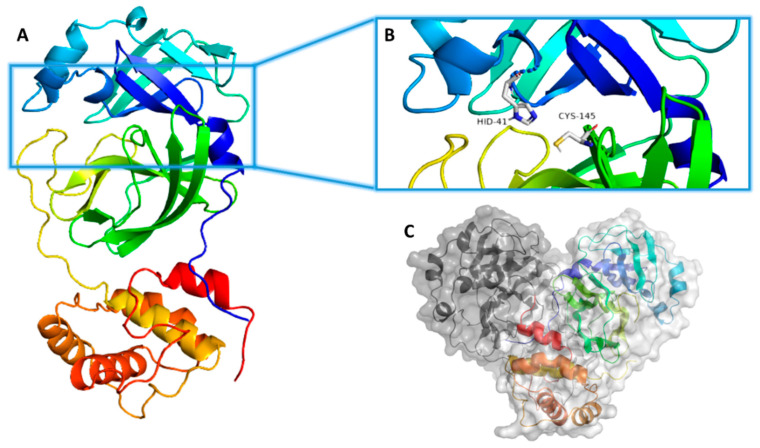
(**A**) Monomeric structure of SARS CoV-2 M^Pro^ (PDB: 6LU7) showing its three main domains (I, II, and III; blue, green, and orange, respectively). (**B**) M^Pro^ active site showing the catalytic dyad (HIS41–CYS145). (**C**) The dimeric active form of SARS CoV-2 M^Pro^.

Structurally, M^Pro^ occurs in a dimeric arrangement (Figure 1), and its hydrolytic activity depends on this structural assembly [6,7]. Each monomer comprises three domains (I, II, and III), where the catalytic active site occurs in a junction between domains I and II. On the other hand, domain III mediates the enzyme dimerization to reach its final active form [7,8] (Figure 1). The enzyme active site has a conserved catalytic dyad consisting of both HIS-41 and CYS-145, via which the enzyme hydrolyzes protein peptide bonds. Any mutations or modifications that occur in these two catalytic residues eventually lead to a complete loss of enzyme hydrolytic activity [9]. Recently, a number of covalent inhibitors (i.e., able to form a covalent bond with CYS-145) have been reported [10], while noncovalent competitive inhibitors are underexplored [11].

**Figure 2:**
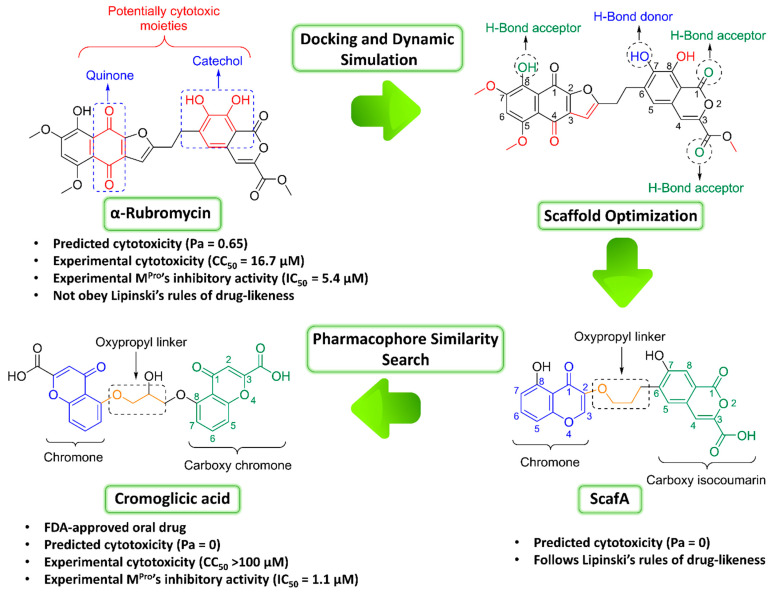
The general outline of the present investigation illustrating the main steps starting from α-rubromycin and eventually reaching cromoglicic acid.

Natural products have offered many anti-SARS CoV-2 agents so far. Among them, ivermectin and artemisinin have shown promising clinical efficacy [12,13,14,15,16]. Consequently, we aimed to extend our investigation of microbial natural products to find potential anti-SARS CoV-2 drug candidates by targeting its M^Pro^. The fermentation products of *Streptomyces collinus* ATCC19743 afforded the quinone antibiotic α-rubromycin as a major metabolite [17]. Testing of this metabolite against the SARS CoV-2 M^Pro^ revealed that it has great potential as an inhibitor. However, it also showed significant toxicity toward normal human cell lines. Accordingly, we decided to modify its core structure (i.e., scaffold hopping) with the aid of molecular docking, molecular dynamics simulation, and binding free energy calculation to get a more appropriate scaffold in terms of cellular toxicity. Furthermore, we applied a pharmacophore-based virtual screening of FDA-approved drugs using this modified scaffold as a template to find structurally similar candidates that can inhibit the M^Pro^ catalytic activity in vitro, thus allowing it to be repurposed as a transient therapeutic agent against COVID-19, just like the previous FDA-approved drug ivermectin [12,13]. The outline of the applied strategy in this communication is depicted in Figure 2.

## 2. Results

As part of our continuous effort to find possible natural product-based therapeutics against SARS CoV-2, we investigated a small microorganism-derived metabolite internal library against the viral M^Pro^. One of the tested molecules was α-rubromycin which was recovered from *Streptomyces collinus* fermentation broth as a major metabolite. Previously, this compound, together with its congeners, was found to exhibit considerable anticancer activity and antiviral potential toward human immunodeficiency virus (HIV) [18,19]. Testing this compound against SARS CoV-2 M^Pro^ showed a promising outcome (IC_50_ = 5.4 ± 0.2 µM and *K*_i_ = 3.22 ± 0.3 µM). However, its significant cytotoxicity toward normal cell lines (CC_50_ 16.7 ± 0.3 µM) was a profound problem. In addition to being a toxic natural product, α-rubromycin also has poor drug-like properties. According to Lipinski’s and Veber’s rules of drug-likeness [20,21], this compound is unsuitable (i.e., has a molecular weight >500, has >10 oxygen atoms, and its topological polar surface area is >150 Å^2^).

**Figure 3:**
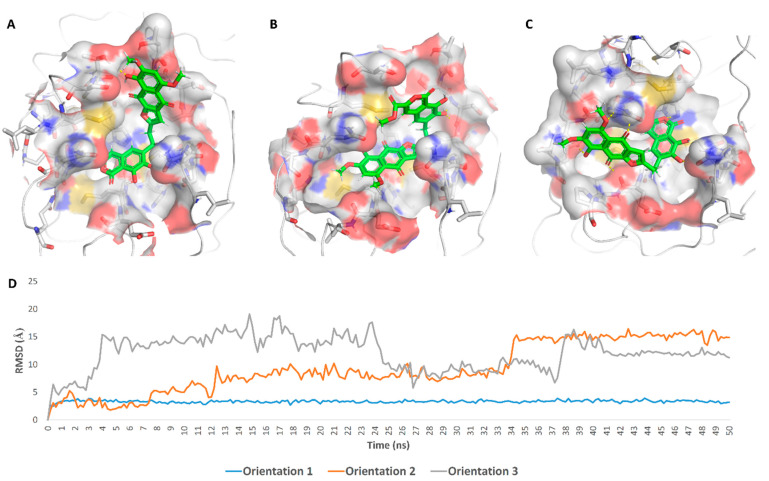
Binding orientations of α-rubromycin inside the M^Pro^ active site (orientations 1–3, **A**–**C** respectively), and their average RMSDs over 50 ns of MDS (**D**).

**Figure 4:**
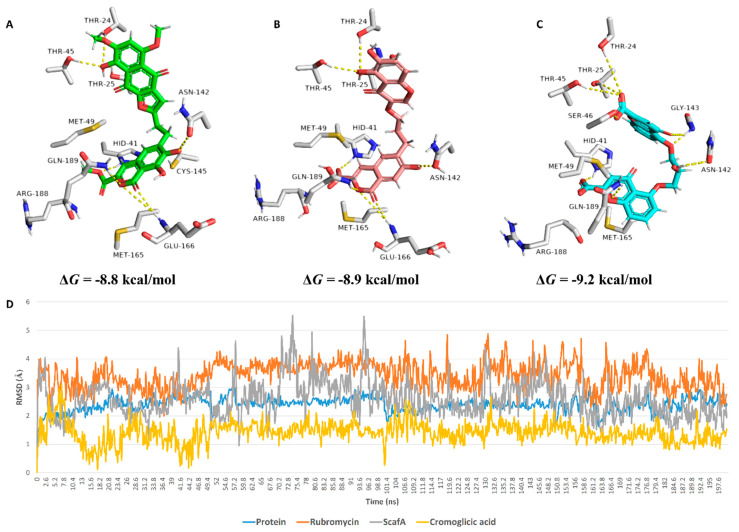
Binding modes of (**A**) α-rubromycin, (**B**) ScafA, and (**C**) cromoglicic acid inside the M^Pro^ active site. (**D**) RMSDs of their docking pose over 200 ns of MDS.

These findings encouraged us to initiate scaffold hopping trials on α-rubromycin along with a pharmacophore-based virtual screening to find promising candidates with enhanced competitive noncovalent enzyme inhibitory activity and minimal toxicity. Docking α-rubromycin into the M^Pro^ active site resulted in seven different docking poses with three main orientations inside the active site (Figure 3). We subjected all generated poses to 50 ns MDS experiments to distinguish between the correct poses and the decoy ones. Only the top-ranked poses (i.e., first three poses) representing the first orientation remained stable during the course of simulation with an average RMSD of 3.3 Å. The other poses were remarkably unstable (Figure 3) and, hence, the top-ranked orientation of α-rubromycin inside M^Pro^ was selected to study its binding mode. α-Rubromycin in this orientation was able to occupy the active site forming four H-bonds with four different amino-acid residues. The methyl ester moiety of the isocoumarin part was perfectly impeded in a binding cavity consisting of HIS-41, MET-49, and ASP-187, H-bonded to HIS-41. Moreover, one of the phenolic hydroxyl groups of the isocoumarin was H-bonded to ASN-142, while the isocoumarin ring system itself was involved in a hydrophobic interaction with both HIS-41 and MET-165. The other part of the molecule (i.e., the naphthazarin ring) was H-bonded to THR-24, THR-25, and THR-45 through its phenolic hydroxyl group (Figure 4 and Table 1).

**Figure 5:**
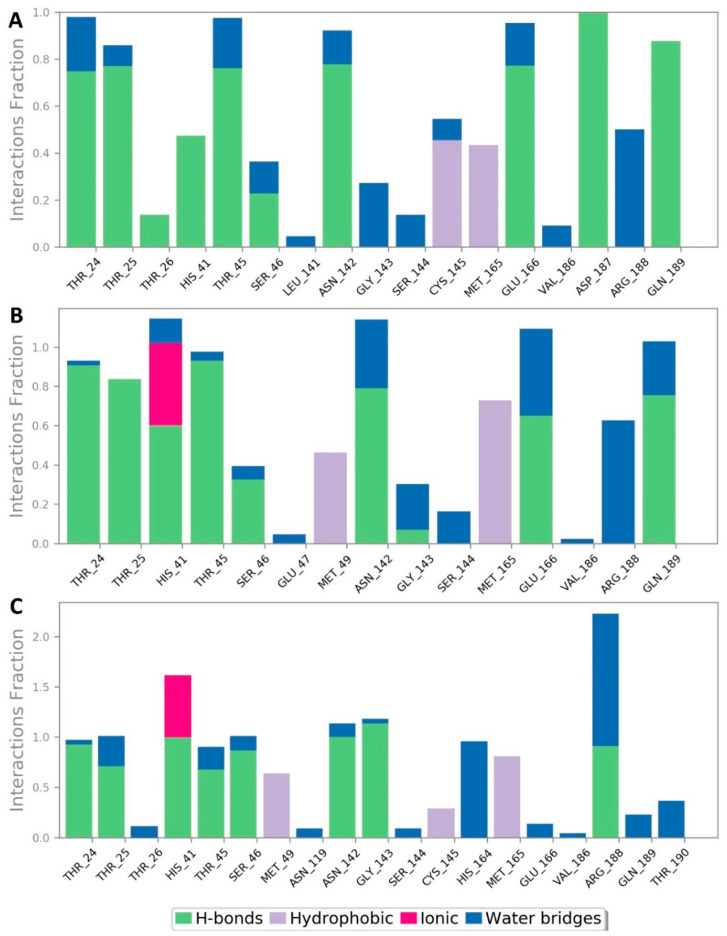
Protein–ligand contacts inside the M^Pro^ active sites over 200 ns of MDS: (**A**) α-rubromycin, (**B**) ScafA, and (**C**) cromoglicic acid.

During a long MDS experiment (200 ns), this binding mode remained stable with an average RMSD of 2.21 Å from the reference orientation and a calculated binding free energy (Δ*G*) of −8.8 kcal/mol. In addition, the generated H-bonds between α-rubromycin and the active site residues remained conserved till the end of MDS. Ligand–protein interaction during the MDS (Figure 5) showed that there were also two important water bridge interactions (>0.3 interaction fraction) between GLY-143 and ARG-188, and the hydroxyl and carboxylate groups of the molecule’s isocoumarin moiety, respectively, which contributed to the stability of α-rubromycin inside the M^Pro^ active site. This binding mode analysis justified the α-rubromycin inhibitory activity toward M^Pro^. Hence, finding a convergent scaffold with reduced cytotoxicity and improved drug-likeness will be highly useful as a starting point for developing new COVID-19 therapeutics.

To do so, we visually inspected the α-rubromycin scaffold looking for the structural elements that may be responsible for its apparent cytotoxicity. We found that the catechol moiety of the molecule’s isocoumarin part is among the highly reactive functional groups and has been reported to induce broad-spectrum toxicity [22] due to its ability to inactivate protein thiol groups [23,24]. Moreover, the quinone moiety of the molecule’s naphthazarin part has been also reported as a potential reactive and toxic chemical species [25,26,27]. Both reactive moieties are common in highly toxic molecules that have been identified as pan assay interference compounds (PAINS). Thus, such chemical entities should be excluded during high-throughput screening campaigns [28].

According to this information, we designed a modified scaffold (ScafA) from α-rubromycin to improve its drug-likeness and reduce its cytotoxicity. Accordingly, α-rubromycin scaffold optimization was achieved by removing the nonessential structural element (i.e., not involved in any interactions with the M^Pro^ active site) to reduce its molecular weight and reduce the number of oxygen atoms. As shown in Figure 2, only one hydroxyl group of the catechol moiety was involved as an H-bond donor in the interaction of α-rubromycin inside the M^Pro^ active site. With regard to the two oxygen atoms of the quinone moiety, neither was involved in any direct interactions with the active site residues. However, the ketone group at C-1 is able to form an intramolecular H-bond with the hydroxyl group at C-8, making it a stronger H-bond acceptor. Consequently, the removal of the noninteracting hydroxyl group from the catechol moiety and replacing the less important ketone of the quinone moiety with an oxygen atom led to an improved scaffold with the same molecular interaction inside the M^Pro^ active site but with likely much lower cellular toxicity. Further removal of the noninteracting parts in the α-rubromycin scaffold eventually led to the proposed optimized scaffold (ScafA) with minimal predicted cytotoxicity and better drug-likeness properties (Figure 2).

In silico neural network-based cellular toxicity prediction showed that ScafA was noncytotoxic in normal cell lines (Pa = 0). In contrast, α-rubromycin was predicted to be cytotoxic toward normal cell lines (Pa = 0.65), and this cytotoxicity was also observed experimentally (IC_50_ = 16.7 µg/mL, Figure 2). Docking ScafA into the M^Pro^ active site resulted in a binding mode almost identical to that of α-rubromycin (Figure 4, Table 1) except for the carboxylate moiety, which interacted with HIS-41 through a salt bridge. Moreover, it achieved binding stability and Δ*G* over 200 ns of MDS which was convergent to that of α-rubromycin (Figure 4).

According to chemical databases (e.g., chemspider, Pubchem, Reaxys, and Scifinder), ScafA is a novel scaffold, and obtaining this compound synthetically will be an interesting starting point for the future. However, faster approaches are highly recommended at this critical time to find potential COVID-19 therapeutics. Consequently, we utilized ScafA as a template in a similarity search for the most similar scaffolds in terms of pharmacophoric features among FDA-approved drugs. We used two independent software for this purpose, Ftrees and SwissSimilarity [29,30], along with the updated database of FDA-approved drugs hosted in the Zinc database (http://zinc.docking.org/substances/subsets/fda/?page=1 accessed on 12 March 2021). Interestingly, cromoglicic acid (i.e., cromolyn) was the top retrieved hit with high similarity scores of 0.84 and 0.79, respectively. This FDA-approved oral medication is mainly used for the long-term management of bronchial asthma by acting as a mast cell stabilizer [31]. Other hits retrieved using either ScafA or α-rubromycin as a template shared very low similarity (≤0.3; Table S1, Supplementary Materials).

The structure of cromoglicic acid is highly similar to that of ScafA and, to some extent, to that of α-rubromycin (similarity score = 0.56). As shown in Figure 2, the hydroxy chromone moiety of ScafA is linked to another carboxyl isocoumarin moiety through an oxypropyl linker, while, in cromoglicic acid, a carboxy chromone moiety is linked to another one via a hydroxy oxypropyl linker to produce a symmetric molecule. The carboxy chromone group of cromoglicic acid is considered an isostere to the carboxyl isocoumarin of ScafA. Hence, the main differences between the two scaffolds are (i) the structure symmetry and (ii) the orientation of attachment to the oxypropyl linker (Figure 2). Docking of cromoglicic acid into the M^Pro^ active site revealed that it could achieve a binding mode similar to that of both α-rubromycin and ScafA (Figure 4) except for the interaction with GLU-166, which was absent in the case of cromoglicic acid, instead able to interact with GLY-143 unlike both α-rubromycin and ScafA (Figure 4 and Table 1). These interactions remained intact throughout the MDS, where the whole molecule achieved better stability, lower Δ*G*, and less deviation from the initial docking pose (average RMSD ~1.4 Å, Figure 4). This greater binding stability could explain the higher inhibitory activity of cromoglicic acid toward M^Pro^ catalytic activity (IC_50_ = 1.1 ± 0.2 µM and *K*_i_ = 0.68 ± 0.1 µM). Moreover, it did not show a cytotoxic effect on human fibroblasts at the highest tested concentration (CC_50_ > 100 µM).

**Figure 6:**
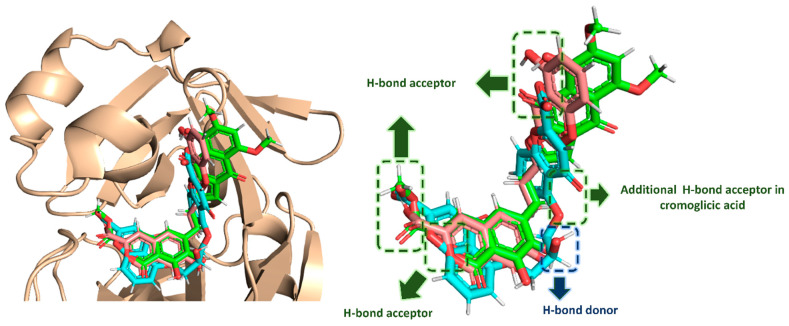
Docking pose alignment of α-rubromycin (green color), ScafA (red color), and cromoglicic acid (cyan color). This alignment shows that the three compounds have a good alignment and share the same pharmacophoric features.

Upon the alignment of docking poses of the three scaffolds (i.e., α-rubromycin, ScafA, and cromoglicic acid), it was obvious that they were perfectly aligned to each other sharing the same pharmacophoric features (Figure 6), which consisted of (i) three H-bond acceptors from HIS-41, GLN-189, THR-24, THR-25, and THR-45, and (ii) a H-bond donor to ASN-142. Moreover, the cromoglicic acid scaffold showed an additional H-bond acceptor from GLY-143 (Figure 6) that could be responsible for its higher binding efficiency and, hence, its higher inhibitory activity.

## 3. Discussion

Despite the recent development of different vaccines as a prophylactic measure against SARS-CoV-2 in late 2020 and early 2021, searching for a proper antiviral agent is crucial, particularly in countries with weak economies. On the other hand, repurposing already available medications has proven its efficacy during the pandemic [32,33,34]. Even if specific SARS CoV-2 treatments become available, reusing already available medicines is still needed to manage this complicated disease. SARS CoV-2 protein structures have been well characterized; hence, myriad small-molecule libraries can be screened against these molecular targets to find suitable candidates for further development. Natural products account for an important portion of these small molecules, thus being a promising pipeline for discovering cost-effective antiviral candidates. Even in natural products with poor drug-like properties, their unprecedented scaffolds can be optimized with the help of the rapidly developing in silico and molecular modeling tools. Herein, from a small in-house microbial natural product library, α-rubromycin, a *Streptomyces*-derived antibiotic, was able to inhibit the catalytic activity of one of the key SARS CoV-2 enzymes (i.e., M^Pro^) with a promising IC_50_ value. However, it displayed cellular toxicity, which is a big concern in drug development. In addition, its calculated drug-like properties according to Lipinski’s and Veber’s rules were obviously poor. With the aid of molecular docking and a subsequent dynamic simulation, we proposed the binding mode of α-rubromycin inside the M^Pro^ active site. In turn, we were able to extract the main pharmacophoric features that contributed to its interaction with M^Pro^. Consequently, hopping of the α-rubromycin scaffold by removing the nonessential structural elements led to a novel drug-like scaffold (ScafA) without any predicted cellular toxicity. However, reaching this novel compound synthetically is a complex and time-consuming process that we have already begun. Nonetheless, we used this scaffold (i.e., ScafA) as a template to look for an FDA-approved drug with similar pharmacophoric features. By utilizing pharmacophore-based virtual screening software, we discovered cromoglicic acid (also known as cromolyn) as a symmetric scaffold comparable to ScafA with almost the same pharmacophoric features. Consequently, it has the potential to inhibit the catalytic activity of M^Pro^ just like α-rubromycin. Subsequent docking, molecular dynamics, and in vitro testing supported this assumption. The results showed that cromoglicic acid was 4.5 times more potent than α-rubromycin owing to its better interaction inside the enzyme active site. In addition, it showed no cytotoxicity toward human fibroblasts at the highest concentration tested (CC_50_ > 100 µg/mL). Despite the IC_50_s of both α-rubromycin and cromoglicic acid being higher than that of the reference covalent inhibitor (i.e., GC376), they are considered potent inhibitors according to recently reported noncovalent M^Pro^ inhibitors with IC_50_s ranging from 0.66 to 10.96 µM that were active against SARS CoV-2 in vitro [35,36]. Being a safe FDA-approved drug, the scaffold of cromoglicic acid is promising for further optimization to produce far more potent inhibitors with very high potential as specific therapeutic candidates.

The sodium salt of cromoglicic acid is commercially available as an eye drop, nasal spray, and oral suspension. It mainly acts as a mast cell stabilizer by preventing the release of common inflammatory mediators such as histamine [31]. This medication is used primarily to manage asthma attacks [37,38] together with corticosteroid-based drugs. Additionally, it is very effective in alleviating allergic rhinitis and conjunctivitis symptoms. Moreover, its oral preparation is prescribed for the treatment of mastocytosis [31]. A number of recent reports revealed that mast cells have a direct role in the development of COVID-19 complications. Hence, using mast cell stabilizers such as cromoglicic acid could also play a crucial role in reducing the severity of the COVID-19 inflammatory phase, particularly if taken early in the course of the infection (3–5 days) [39,40,41].

Accordingly, cromoglicic acid could have the potential to fight COVID-19 through a dual mode of action: (i) suppressing the severity of its cytokine storm and (ii) fighting the virus itself by inhibiting one of its critical hydrolytic enzymes (i.e., M^Pro^).

It is worth noting that a number of M^Pro^ inhibitors are now under clinical investigation (e.g., the covalent inhibitor compound 9 “NCT04535167” and the noncovalent inhibitor baicalin “NCT03830684”) [35,42].

## 4. Materials and Methods

### 4.1. Isolation of α-Rubromycin

The seed culture of *S. collinus* ATCC19743 was prepared in ISP2 medium and fermented on shaker incubator for 3 days at 30 °C and 180 rpm. Then, the seed culture was used for large-scale 3 L production medium (ISP2) on a shaker incubator for 7 days at 30 °C and 180 rpm. The culture was centrifuged (3000 rpm for 10 min), and the cell mass was washed and extracted with MeOH (4 × 250 mL). The successive MeOH extracts were combined and concentrated under vacuum to 200 mL and successively fractionated with *n*-hexane (3 × 200 mL) to remove the fats and for CH_2_Cl_2_ (3 × 200 mL) compound isolation. RP-HPLC purified this CH_2_Cl_2_ fraction (40 mg) (sunfire ^TM^, prep C18, 5 µm, 10 × 250 mm) using a gradient of MeOH in H_2_O (50–100% over 30 min) followed by 100% MeOH for 10 min at a flow rate of 1.25 mL·min^−1^ to afford the pure α-rubromycin (3.5 mg; purity > 90%) which was confirmed using HRMS and advanced NMR analyses. This isolated form of α-rubromycin was used for the rapid screening step against M^Pro^; however, but for the detailed in vitro assays (e.g., IC_50_ calculation), α-rubromycin was purchased from Enzo Life Sciences (Product Code. ALX-380-067-M005, Enzo Life Sciences, Exeter, UK) to ensure >98% purity.

### 4.2. In-Vitro Assays

#### 4.2.1. M^Pro^ Inhibition

In vitro enzyme inhibition assays were performed using the commercially available SARS-CoV-2 main protease assay kit (Catalog #: 79955-1, BPS Bioscience, Inc., Allentown, PA, USA) and according to the manufacturer’s protocol. GC376 was used as a reference standard inhibitor (IC_50_ = 0.22 µM). Substrate cleavage by M^Pro^ produced fluorescence which was observed at 460 nm and 360 nm (emission and excitation wavelengths, respectively) using a Tecan Spark microplate reader (Tecan, Männedorf, Switzerland). Briefly, 10 µL of test compounds at different concentrations were added into a 96-well plate, followed by pipetting 30 µL of the diluted protease (15 µg/mL). The mixtures were incubated for 30 min at room temperature, and then 10 µL of the substrate was dissolved in the reaction buffer and added to reach a 50 µL final volume and 40 µM final concentration. The reaction mixture was then incubated for 4 h at 20 °C followed by measuring the produced fluorescence using a TECAN spark (Tecan, Männedorf, Switzerland) microplate-reading fluorimeter. The inhibition constant (*K*_i_) values for each inhibitor were determined according to the manufacturer protocol, where the rate of substrate utilization, using 2 mM of the tested enzyme and 0–250 µM of the substrate, was monitored in increasing amounts of inhibitor (0–50 µM).

#### 4.2.2. MTT Cytotoxicity Assay

To determine the half-maximal cell toxicity concentration (CC_50_) to assess the compound toxicity, we prepared stock solutions of the test compounds in 10% DMSO with ddH_2_O and further diluted with DMEM to the working solutions. The cytotoxic effect of the tested compounds was evaluated in normal human adult dermal fibroblasts (HDFa) (Sigma-Aldrich, Dorset, UK) using the previously reported 3-(4,5-dimethylthiazol-2-yl)-2,5-diphenyltetrazolium bromide (MTT) method [43] with slight adjustments. Briefly, 100 µL cells/well at a density of 3 × 10^5^ cells/mL were placed in 96-well plates and then incubated in 5% CO_2_ for 24 h at 37 °C. After 24 h, different concentrations of the test compound(s) were added to the cells in triplicate. After another 24 h, the supernatant was removed, and cells were washed with sterile 1× PBS three times. Then, 20 µL of 5 mg/mL MTT stock solution was added to each well, and the plate was incubated at 37 °C for 4 h. The produced formazan crystals were dissolved in 200 µL of acidified isopropanol (0.04 M HCl in absolute isopropanol). After that, we measured the absorbance of formazan solutions at λ_max_ 540 nm using a BioTek Lx800 microplate reader (BioTek Instrument Ltd., Bedfordshire, UK). The cytotoxicity percentage compared to the control cells was determined using the following Equation (1):

(1) $$\%\;\mathrm{cytotoxicity}=\frac{\left(\mathrm{absorbance}\;\mathrm{of}\;\mathrm{cells}\;\mathrm{without}\;\mathrm{treatment}-\mathrm{absorbance}\;\mathrm{of}\;\mathrm{cells}\;\mathrm{with}\;\mathrm{treatment}\right)\times 100}{\mathrm{absorbance}\;\mathrm{of}\;\mathrm{cells}\;\mathrm{without}\;\mathrm{treatment}}.$$

The produced plot of percentage cytotoxicity versus sample concentrations was then used to calculate the CC_50_s.

### 4.3. In Silico Investigation

#### 4.3.1. Ensemble Docking

AutoDock Vina software was used in all molecular docking experiments [44]. All compounds were docked against the M^Pro^ crystal structure (PDB codes: 6LU7) [45]. The binding site was determined according to the enzyme’s co-crystallized ligand. The active site of M^Pro^ is relatively flexible [8,46], and, to account for this flexibility, we used MDS-derived conformers sampled every 10 ns for docking experiments (i.e., ensemble docking) [8,46]. Subsequently, we ranked the resulting top hits according to their calculated binding energies. Docking poses were analyzed and visualized using Pymol software [44].

#### 4.3.2. Molecular Dynamics Simulation

Desmond v. 2.2 software was used for performing MDS experiments [47,48,49]. This software applies the OPLS force field. Protein systems were built using the System Builder option, where the protein structure was embedded in an orthorhombic box of TIP3P water together with 0.15 M Na^+^ and Cl^−^ ions in 20 Å solvent buffer. Afterward, the prepared systems were energy minimized and equilibrated for 10 ns.

Desmond software automatically parameterizes inputted ligands during the system building step according to the OPLS force field. For simulations performed by NAMD [50], the parameters and topologies of the compounds were calculated either using the Charmm27 force field with the online software Ligand Reader and Modeler (http://www.charmm-gui.org/?doc=input/ligandrm, accessed on 16 April 2021) [51] or using the VMD plugin Force Field Toolkit (ffTK). Afterward, the generated parameters and topology files were loaded to VMD to readily read the protein–ligand complexes without errors and then conduct the simulation step.

#### 4.3.3. Binding Free Energy Calculations

Binding free energy calculations (Δ*G*) were performed using the free energy perturbation (FEP) method [51]. This method was described in detail in the recent article by Kim and coworkers [51]. Briefly, this method calculates the binding free energy Δ*G*_binding_ according to the following equation: Δ*G*_binding_ = Δ*G*_Complex_ − Δ*G*_Ligand_.

The value of each Δ*G* is estimated from a separate simulation using NAMD software. Interestingly, all input files required for simulation by NAMD can be papered by using the online website Charmm-GUI (https://charmm-gui.org/?doc=input/afes.abinding, accessed on 16 April 2021). Subsequently, we can use these files in NAMD to produce the required simulations using the FEP calculation function in NAMD. The equilibration was achieved in the NPT ensemble at 300 K and 1 atm (1.01325 bar) with Langevin piston pressure (for ″Complex″ and ″Ligand″) in the presence of the TIP3P water model. Then, 10 ns FEP simulations were performed for each compound, and the last 5 ns of the free energy values was measured for the final free energy values [51]. Finally, the generated trajectories were visualized and analyzed using VMD software. It worth noting that Ngo and coworkers in their recent benchmarking study found that the FEP method of determination of Δ*G* was the most accurate method in terms of predicting M^Pro^ inhibitors [52].

#### 4.3.4. Drug-Likeness Analysis

Drug-like properties of the studied compounds were predicted by the commercially available software LigandScout 4.3 [53]. A list of SMILES codes of these compounds was prepared and submitted to the software to perform the drug-likeness calculations (e.g., molecular weight, hydrogen bond donors, hydrogen bond acceptors, number of rotatable bonds, topological polar surface area, and logP). As a final result, we checked if these calculated parameters for each compound followed Lipiniski’ and Vebers’ rules of drug likeness [20,21].

#### 4.3.5. Toxicity Prediction

Cytotoxicity toward normal cell lines was predicted using CLC-Pred (Cell Line Cytotoxicity Predictor). Prediction is dependent on PASS (Prediction of Activity Spectra for Substances) technology (http://www.way2drug.com/PASSonline, accessed on 21 April 2021), and the training set was shaped on the basis of data on cytotoxicity obtained from ChEMBLdb (version 23) (https://www.ebi.ac.uk/chembldb/, accessed on 16 April 2021) [54]. After submitting the SMILES code of each compound, the software gives the predicted cytotoxicity arranged according to the cell line type and their activity scores (probability of being active score; Pa).

#### 4.3.6. Pharmacophore-Based Screening

Pharmacophore-based screening was performed using two independent software. The first was SwissSimilarity (http://www.swisssimilarity.ch/, accessed on 16 April 2021) [30]. The SMILES code of the query compound was first submitted, and the FDA-approved database was selected to apply the screening. The computation method was applied according to the pharmacophore features of the input compound (i.e., spectrophores). The retrieved results were ranked according to the similarity degree (from 0 to 1).

The second used software was Ftrees (http://www.biosolveit.de/FTrees, accessed on 16 April 2021) [29]. Molecules similar to the query molecule were screened against FDA-approved drugs hosted in the Zinc database (http://zinc.docking.org/substances/subsets/fda/?page=1, accessed on 3 April 2021). This software applies a different searching approach based on a complex feature tree instead of linear fingerprint depictions [55]. Unlike fingerprint-based similarity search, the minimum FTrees similarity score between the query and the target molecules (similarity threshold) was set to a fixed value of 0.8. The FTrees visual similarities output is a particular similarity score for each query pair.

## 5. Conclusions

In conclusion, our scaffold hopping approach applied in this investigation achieved two goals: (1) leading to the discovery of a promising and novel drug-like M^Pro^ inhibitor candidate (ScafA), and (2) leading to the discovery of a safe and promising FDA-approved drug as a potent M^Pro^ inhibitor, which has already shown significant benefits in managing COVID-19 inflammatory complications. Additionally, it highlighted microbial natural products as a central source of unprecedented structural motifs that have high potential to be developed into a wide range of effective therapeutics.

## Figures and Tables

**Table 1:** Docking scores and Δ*G*s of α-rubromycin, ScafA, and cromoglicic acid, along with their interactions inside the M^Pro^ active site.

Compound	Docking SCORE	ΔG *	Interactions		
			H-Bonding	Water Bridges	Hydrophobic
**α-Rubromycin**	−8.2 kcal/mol	−8.8 kcal/mol	THR-24, THR-25, HIS-41, ASN-142, GLU-166, GLN-189	ARG-188	MET-49, CYS-145, MET-165
**ScafA**	−8.2 kcal/mol	−8.9 kcal/mol	THR-24, THR-25, HIS-41 **, ASN-142, GLU-166, GLN-189	GLU-166, ASN-142, ARG-188	MET-49, CYS-145, MET-165
**Cromoglicic acid**	−8.3 kcal/mol	−9.2 kcal/mol	THR-24, THR-25, HIS-41 **, SER-46, ASN-142, GLY-143, GLN-189	HIS-164, ARG-188	MET-49, CYS-145, MET-165

* Δ*G* is the binding free energy calculated using the FEP method; ** Salt bridge interaction.

## Data Availability

The data presented in this study are available on request from the corresponding author.

## Supplementary Materials

The following are available online at https://www.mdpi.com/article/10.3390/ph14060541/s1, Table S1: Results of ScafA similarity search in the FDA-approved drugs.

## References

[1] Sayed A.M., Khattab A.R., AboulMagd A.M., Hassan H.M., Rateb M.E., Zaid H., Abdelmohsen U.R. (2020). Nature as a treasure trove of potential anti-SARS-CoV drug leads: A structural/mechanistic rationale. RSC Adv..

[2] Adamson C.S., Chibale K., Goss R.J., Jaspars M., Newman D.J., Dorrington R.A. (2021). Antiviral drug discovery: Preparing for the next pandemic. Chem. Soc. Rev..

[3] Zhang L., Lin D., Sun X., Curth U., Drosten C., Sauerhering L., Becker S., Rox K., Hilgenfeld R. (2020). Crystal structure of SARS-CoV-2 main protease provides a basis for design of improved α-ketoamide inhibitors. Science.

[4] Ullrich S., Nitsche C. (2020). The SARS-CoV-2 main protease as drug target. Bioorg. Med. Chem. Lett..

[5] Dai W., Zhang B., Jiang X.M., Su H., Li J., Zhao Y., Xie X., Jin Z., Peng J., Liu F. (2020). Structure-based design of antiviral drug candidates targeting the SARS-CoV-2 main protease. Science.

[6] Behnam M.A. (2021). Protein structural heterogeneity: A hypothesis for the basis of proteolytic recognition by the main protease of SARS-CoV and SARS-CoV-2. Biochimie.

[7] Pavlova A., Lynch D.L., Daidone I., Zanetti-Polzi L., Smith M.D., Chipot C., Kneller D.W., Kovalevsky A., Coates L., Golosov A.A. (2021). Inhibitor binding influences the protonation states of histidines in SARS-CoV-2 main protease. Chem. Sci..

[8] Sayed A.M., Alhadrami H.A., El-Gendy A.O., Shamikh Y.I., Belbahri L., Hassan H.M., Abdelmohsen U.R., Rateb M.E. (2020). Microbial natural products as potential inhibitors of SARS-CoV-2 main protease (M^pro^). Microorganisms.

[9] Chang H.P., Chou C.Y., Chang G.G. (2007). Reversible unfolding of the severe acute respiratory syndrome coronavirus main protease in guanidinium chloride. Biophys. J..

[10] Mondal D., Warshel A. (2020). Exploring the Mechanism of Covalent Inhibition: Simulating the Binding Free Energy of α-Ketoamide Inhibitors of the Main Protease of SARS-CoV-2. Biochemistry.

[11] Zhou Q.A., Kato-Weinstein J., Li Y., Deng Y., Granet R., Garner L., Liu C., Polshakov D., Gessner G., Watkins S. (2020). Potential therapeutic agents and associated bioassay data for COVID-19 and related human coronavirus infections. ACS Pharmacol. Transl. Sci..

[12] Caly L., Druce J.D., Catton M.G., Jans D.A., Wagstaff K.M. (2020). The FDA-approved drug ivermectin inhibits the replication of SARS-CoV-2 in vitro. Antiviral Res..

[13] Sharun K., Dhama K., Patel S.K., Pathak M., Tiwari R., Singh B.R., Sah R., Bonilla-Aldana D.A., Rodriguez-Morales A.J., Leblebicioglu H. (2020). Ivermectin, a new candidate therapeutic against SARS-CoV-2/COVID-19. Ann. Clin. Microbiol. Antimicrob..

[14] Behera P., Patro B.K., Singh A.K., Chandanshive P.D., Pradhan S.K., Pentapati S.S.K., Batmanabane G., Mohapatra P.R., Padhy B.M., Bal S.K. (2021). Role of ivermectin in the prevention of SARS-CoV-2 infection among healthcare workers in India: A matched case-control study. PLoS ONE.

[15] Cao R., Hu H., Li Y., Wang X., Xu M., Liu J., Zhong W. (2020). Anti-SARS-CoV-2 potential of artemisinin in vitro. ACS Infect. Dis..

[16] Uckun F.M., Saund S., Windlass H., Trieu V. (2021). Repurposing Anti-Malaria Phytomedicine Artemisinin as a COVID-19 Drug. Front. Pharmacol..

[17] Atkinson D.J., Brimble M.A. (2015). Isolation, biological activity, biosynthesis and synthetic studies towards the rubromycin family of natural products. Nat. Prod. Rep..

[18] Goldman M.E., Salituro G.S., Bowen J.A., Williamson J.M., Zink D.L., Schleif W.A., Emini E.A. (1990). Inhibition of human immunodeficiency virus-1 reverse transcriptase activity by rubromycins: Competitive interaction at the template. primer site. Mol. Pharmacol..

[19] Metsä-Ketelä M., Ylihonko K., Mäntsälä P. (2004). Partial activation of a silent angucycline-type gene cluster from a rubromycin β producing Streptomyces sp. PGA64. J. Antibiot..

[20] Lipinski C.A., Lombardo F., Dominy B.W., Feeney P.J. (1997). Experimental and computational approaches to estimate solubility and permeability in drug discovery and development settings. Adv. Drug Delivery Rev..

[21] Veber D.F., Johnson S.R., Cheng H.Y., Smith B.R., Ward K.W., Kopple K.D. (2002). Molecular properties that influence the oral bioavailability of drug candidates. J. Med. Chem..

[22] Bukowska B., Kowalska S. (2004). Phenol and catechol induce prehemolytic and hemolytic changes in human erythrocytes. Toxicol. Lett..

[23] Metz J.T., Huth J.R., Hajduk P.J. (2007). Enhancement of chemical rules for predicting compound reactivity towards protein thiol groups. J. Comput. Aided Mol. Des..

[24] Huth J.R., Song D., Mendoza R.R., Black-Schaefer C.L., Mack J.C., Dorwin S.A., Ladror U.S., Severin J.M., Walter K.A., Bartley D.M. (2007). Toxicological evaluation of thiol-reactive compounds identified using a la assay to detect reactive molecules by nuclear magnetic resonance. Chem. Res. Toxicol..

[25] Monks T.J., Jones D.C. (2002). The metabolism and toxicity of quinones, quinonimines, quinone methides, and quinone-thioethers. Curr. Drug Metabol..

[26] Liu X.W., Sok D.E. (2003). Identification of alkylation-sensitive target chaperone proteins and their reactivity with natural products containing Michael acceptor. Arch. Pharmacal Res..

[27] Li W.W., Heinze J., Haehnel W. (2005). Site-specific binding of quinones to proteins through thiol addition and addition− elimination reactions. J. Amer. Chem. Soc..

[28] Baell J.B., Holloway G.A. (2010). New substructure filters for removal of pan assay interference compounds (PAINS) from screening libraries and for their exclusion in bioassays. J. Med. Chem..

[29] Rarey M., Dixon J.S. (1998). Feature trees: A new molecular similarity measure based on tree matching. J. Comput. Aided Mol. Des..

[30] Zoete V., Daina A., Bovigny C., Michielin O. (2016). SwissSimilarity: A web tool for low to ultra-high throughput ligand-based virtual screening. J. Chem. Inf. Model..

[31] Flower R.J., Henderson G., Loke Y.K., MacEwan D., Rang H.P. (2018). Rang & Dale’s Pharmacology E-Book.

[32] Singh T.U., Parida S., Lingaraju M.C., Kesavan M., Kumar D., Singh R.K. (2020). Drug repurposing approach to fight COVID-19. Pharmacol. Rep..

[33] Johnson R.M., Vinetz J.M. (2020). Dexamethasone in the management of covid-19. BMJ.

[34] Sayed A.M., Khalaf A.M., Abdelrahim M.E., Elgendy M.O. (2020). Repurposing of some anti-infective drugs for COVID-19 treatment: A surveillance study supported by an in silico investigation. Int. J. Clin. Pract..

[35] Cannalire R., Cerchia C., Beccari A.R., Di Leva F.S., Summa V. (2020). Targeting SARS-CoV-2 Proteases and Polymerase for COVID-19 Treatment: State of the Art and Future Opportunities. J. Med. Chem..

[36] Kitamura N., Sacco M.D., Ma C., Hu Y., Townsend J.A., Meng X., Zhang F., Zhang X., Ba M., Szeto T. (2021). Expedited Approach toward the Rational Design of Noncovalent SARS-CoV-2 Main Protease Inhibitors. J. Med. Chem..

[37] Lambrecht B.N., Hammad H. (2015). The immunology of asthma. Nat. Immunol..

[38] Castillo M., Scott N.W., Mustafa M.Z., Mustafa M.S., Azuara-Blanco A. (2015). Topical antihistamines and mast cell stabilisers for treating seasonal and perennial allergic conjunctivitis. Cochr. Database Syst. Rev..

[39] Sanchez-Gonzalez M.A., Moskowitz D., Issuree P.D., Yatzkan G., Rizvi S.A., Day K. (2020). A Pathophysiological Perspective on COVID-19’s Lethal Complication: From Viremia to Hypersensitivity Pneumonitis-like Immune Dysregulation. Infect. Chemother..

[40] Theoharides T.C. (2021). Potential association of mast cells with coronavirus disease 2019. Ann. Aller. Asth. Immunol..

[41] Afrin L.B., Weinstock L.B., Molderings G.J. (2020). Covid-19 hyperinflammation and post-Covid-19 illness may be rooted in mast cell activation syndrome. Inter. J. Infect. Dis..

[42] (2020). Pfizer Investor Day Features Significant Number of Pipeline Advances for COVID-19 Programs and Across Numerous Therapeutic Areas.

[43] Mosmann T. (1983). Rapid colorimetric assay for cellular growth and survival: Application to proliferation and cytotoxicity assays. J. Immunol. Methods.

[44] Seeliger D., de Groot B.L. (2010). Ligand docking and binding site analysis with PyMOL and Autodock/Vina. J. Comput. Aided Mol. Des..

[45] Jin Z., Du X., Xu Y., Deng Y., Liu M., Zhao Y., Zhang B., Li X., Zhang L., Peng C. (2020). Structure of M pro from SARS-CoV-2 and discovery of its inhibitors. Nature.

[46] Amaro R.E., Baudry J., Chodera J., Demir Ö., McCammon J.A., Miao Y., Smith J.C. (2018). Ensemble docking in drug discovery. Biophys. J..

[47] Bowers K.J., Chow D.E., Xu H., Dror R.O., Eastwood M.P., Gregersen B.A., Klepeis J.L., Kolossvary I., Moraes M.A., Sacerdoti F.D. (2006). Scalable algorithms for molecular dynamics simulations on commodity clusters. Proceedings of the SC’06: Proceedings of the 2006 ACM/IEEE Conference on Supercomputing.

[48] Release S. (2017). 3: Desmond Molecular Dynamics System, DE Shaw Research, New York, NY, 2017.

[49] Schrodinger LLC (2009). Maestro, Version 9.0.

[50] Phillips J.C., Braun R., Wang W., Gumbart J., Tajkhorshid E., Villa E., Chipot C., Skeel R.D., Kalé L., Schulten K. (2005). Scalable molecular dynamics with NAMD. J. Comput. Chem..

[51] Kim S., Oshima H., Zhang H., Kern N.R., Re S., Lee J., Rous B., Sugita Y., Jiang W., Im W. (2020). CHARMM-GUI free energy calculator for absolute and relative ligand solvation and binding free energy simulations. J. Chem. Theory Comput..

[52] Ngo S.T., Tam N.M., Quan P.M., Nguyen T.H. (2021). Benchmark of Popular Free Energy Approaches Revealing the Inhibitors Binding to SARS-CoV-2 Mpro. J. Chem. Inf. Model..

[53] Tutone M., Perricone U., Almerico A.M. (2017). Conf-VLKA: A structure-based revisitation of the Virtual Lock-and-key Approach. J. Mol. Graph. Model..

[54] Lagunin A.A., Dubovskaja V.I., Rudik A.V., Pogodin P.V., Druzhilovskiy D.S., Gloriozova T.A., Filimonov D.A., Sastry N.G., Poroikov V.V. (2018). CLC-Pred: A freely available web-service for in silico prediction of human cell line cytotoxicity for drug-like compounds. PLoS ONE.

[55] Durant J.L., Leland B.A., Henry D.R., Nourse J.G. (2002). Reoptimization of MDL keys for use in drug discovery. J. Chem. Inform. Comput. Sci..

