# Supplementary material for: Scaffold Hopping of α-Rubromycin Enables Direct Access to FDA-Approved Cromoglicic Acid as a SARS-CoV-2 MPro Inhibitor
Source: Pharmaceuticals (Basel). 2021 Jun 5;14(6):541. doi: 10.3390/ph14060541 (PMC8229550; doi:10.3390/ph14060541)
Supplement: Supplementary file 1 [file pharmaceuticals-14-00541-s001.zip › pharmaceuticals-1219888-supplementary.pdf]

Supplementary Material

Scaffold Hopping of α-Rubromycin Enables Direct Access to the FDA-approved Cromoglicic acid as a SARS-CoV-2 M^{Pro} Inhibitor

Hani A. Alhadrami, Ahmed M. Sayed, Heba Alkhatabi, Nabil A. Alhakamy and Mostafa E. Rateb

Table S1. Results of ScafA similarity search in the FDA-approved drugs

Compound	Similarity Score	Structure	Class
Cromoglicic acid	0.84	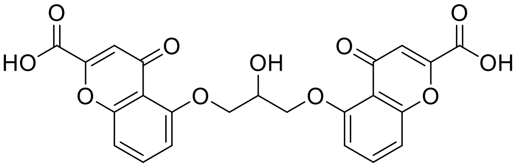	Mast Cell stabilizer*
Nafcillin	0.3	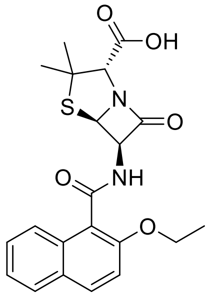	ß-Lactam antibiotic**
Amoxicillin	0.15	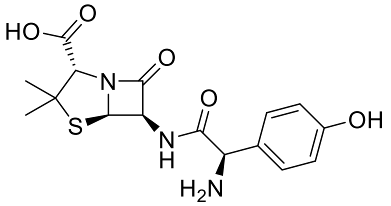	ß-Lactam antibiotic**
Benzylpenicillin	0.11	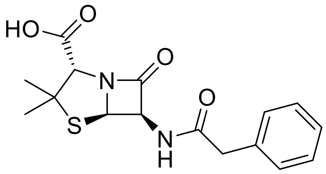	ß-Lactam antibiotic**
Phenoxymethylpenicillin	0.11	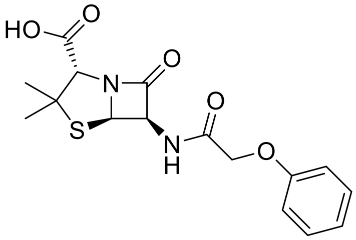	ß-Lactam antibiotic**

Ticarcillin	0.11	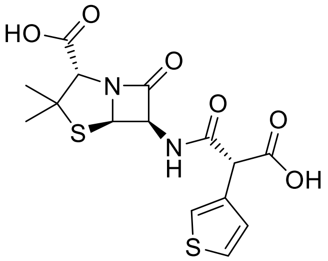	ß-Lactam antibiotic**
Carbenicillin	0.11	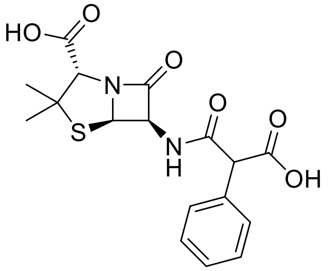	ß-Lactam antibiotic**
Ampicillin	0.11	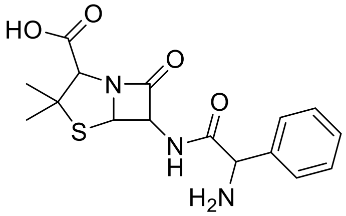	ß-Lactam antibiotic**
Cefalexin	0.11	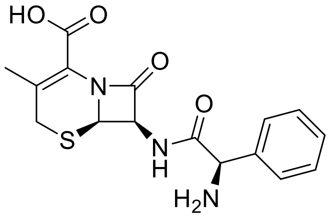	ß-Lactam antibiotic**
Cefuroxime	0.11	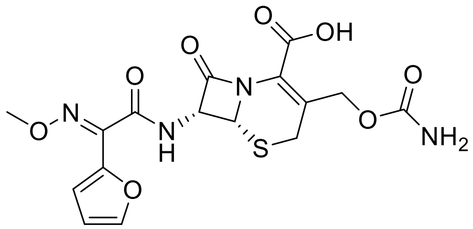	ß-Lactam antibiotic**
Dicoumarol	0.1	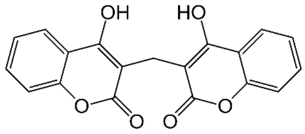	Anticoagulant[#]
Warfarin	0.1	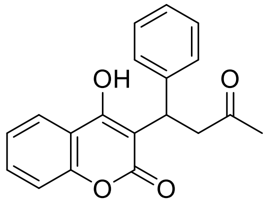	Anticoagulant[#]

Acenocoumarol	0.1	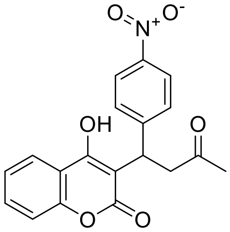	Anticoagulant[#]

*This is the most similar hit. In addition, it showed very low toxicity in vitro

** Frequent uncontrolled use of antibiotics can lead to the emergence of bacterial resistance [1].

[#]Anticoagulants are dangerous drugs with low therapeutic window [2].

References

1. Lucien, M. A. B., Canarie, M. F., Kilgore, P. E., Jean-Denis, G., Fénélon, N., Pierre, M., ... & Ramon-Pardo, P. (2021). Antibiotics and antimicrobial resistance in the COVID-19 era: Perspective from resource-limited settings. *International Journal of Infectious Diseases*, *104*, 250-254.

2. DUFF, I. F., & SHULL, W. H. (1949). Fatal Hemorrhage in Dicumarol® Poisoning. *Journal of the American Medical Association*, *139*(12), 762-766.
